# Effect of Addition of Cr on the Structural Properties of Copper Films on BaTiO_3_ Ceramic Substrates

**DOI:** 10.3390/ma18214851

**Published:** 2025-10-23

**Authors:** Fengtian Shi, Heda Bai, Yuanhao Liao, Jin Li, Xiangli Liu

**Affiliations:** 1 School of Materials Science and Engineering, Harbin Institute of Technology (Shenzhen), Shenzhen 518055, China; 23s155036@stu.hit.edu.cn (F.S.); 20b955008@stu.hit.edu.cn (H.B.); 23s155037@stu.hit.edu.cn (Y.L.); 2 Institute of Special Environments Physical Sciences, Harbin Institute of Technology (Shenzhen), Shenzhen 518055, China; jinli2019@hit.edu.cn

**Keywords:** Cu film, Cr addition, HiPIMS, ceramic substrates, electrical properties, corrosion resistance

## Abstract

In the application of ceramic dielectric filters, to achieve electromagnetic shielding of signals and subsequent integrated applications, it is necessary to carry out metallization treatment on their surfaces. The quality of metallization directly affects the performance of the filter. However, when in use, the filter may encounter harsh environmental conditions. Therefore, the surface-metallized film needs to have strong corrosion resistance to ensure its long-term stability during use. In this paper, Cu films and copper–chromium alloy films were fabricated on Si (100) substrates and BaTiO_3_ ceramic substrates by HiPIMS technology. The effects of different added amounts of Cr on the microstructure, electrical conductivity, and corrosion resistance of the Cu films were studied. The results show that with an increase in Cr content, the preferred orientation of the (111) crystal plane gradually weakens, and the grains of the Cu-Cr alloy film gradually decrease. The particles on the film surface are relatively coarse, increasing the surface roughness of the film. However, after doping, the film still maintains a relatively low surface roughness. After doping with Cr, the resistivity of the film increases with the increase in Cr content. The film–substrate bonding force shows a trend of first increasing and then decreasing with the increase in Cr content. Among them, when the Cr content is 2 at.%, the film–substrate bonding force is the greatest. The Cu-Cr alloy film has good corrosion resistance in static corrosion. With the increase in Cr content, the Tafel slope of the cathode increases, and the polarization resistance R_p_ also increases with the increase in Cr content. After the addition of Cr, both the oxide film resistance and the charge transfer resistance of the electrode reaction of the Cu-Cr alloy film are greater than those of the Cu film. This indicates that the addition of Cr reduces the corrosion rate of the alloy film and enhances its corrosion resistance in a NaCl solution. 2 at.% Cr represents a balanced trade-off in composition. While ensuring the film is dense, uniform, and has good electrical conductivity, the adhesion between the film and the substrate is maximized, and the corrosion resistance of the Cu film is also improved.

## 1. Introduction

To achieve electromagnetic shielding and integration in ceramic dielectric filters, a high-performance metallized layer is required [1,2]. This requires the development of metal films that simultaneously possess high electrical conductivity, strong adhesion to ceramic substrates, and a certain degree of corrosion resistance, especially in coastal areas where devices are exposed to high-salt and high-humidity environments for a long time [3,4].

Copper has long been widely used in the metallization process of filters due to its low resistivity, high thermal conductivity, good resistance to electromigration, and mature process foundation [5,6]. However, copper itself has relatively low mechanical strength and poor oxidation resistance and corrosion resistance. Especially in chlorine-containing environments (such as coastal areas), it is prone to corrosion, which limits its reliability. For this reason, researchers are committed to improving the comprehensive performance of copper films through alloying methods [7,8,9]. Elements such as Fe and Mn are introduced to enhance corrosion resistance: Fe can promote the formation of high-resistance corrosion product layers [10,11] and increase the compactness of the passivation film [12]. However, if the iron content is too high, anodic iron-rich second-phase precipitates will form at the grain boundaries. In corrosive media, these precipitates will preferentially dissolve, leading to intergranular corrosion and significantly reducing the overall corrosion resistance of the alloy [13,14]. The alloying effect of Mn lies in its ability to promote the enrichment of Ni in the protective passivation film to enhance its stability. Additionally, it can generate MnO_2_ to construct an effective physical barrier for the diffusion of corrosive ions (such as Cl^−^), thereby kinetically hindering the corrosion process [13]. However, the effect of Mn is highly dependent on the alloy system and the preparation process [14].

Chromium serves as an ideal alloying element for copper, offering a unique combination of property enhancements. Its primary role is to significantly improve corrosion resistance by facilitating the formation of a protective Cr_2_O_3_ layer on the surface [15,16]. Furthermore, the addition of Cr effectively refines the grain structure of copper films, which enhances mechanical strength [7,17]. Crucially, even with these benefits, the increase in electrical resistivity due to low-concentration Cr doping remains relatively small, allowing the alloy to retain high conductivity [17,18,19,20,21]. This makes Cu-Cr alloys particularly attractive for applications requiring a balance of strength, corrosion resistance, and electrical performance [9,18].

In this study, the high-power pulsed magnetron sputtering (HiPIMS) technique was employed to deposit the films. HiPIMS is characterized by its high ionization rate of the sputtered species [22,23,24]. This high plasma density generates a significant flux of energetic ions that bombard the growing film during deposition. This ion bombardment promotes the formation of a denser, more crystalline microstructure, which is crucial for enhancing electrical conductivity [24,25]. Furthermore, it significantly improves the adhesion strength between the film and the ceramic substrate [26,27,28], making HiPIMS an ideal technique for achieving the dual goals of high conductivity and robust adhesion in metallization applications.

In this study, the basis for choosing 1, 2, and 3 at.% as the Cr addition amount mainly includes the following points: Firstly, at room temperature, the Cu-Cr system is almost immiscible, and the solid solubility of Cr in Cu is extremely low. Only at low content can a stable solid solution be formed and the precipitation of the coarse second phase be avoided [7,29]. Secondly, existing studies have shown that an added amount of approximately 0.5–5 at.% of Cr is the optimal range for achieving both strength and electrical conductivity in Cu-Cr alloys, among which approximately 2 at.% is considered the optimization point [29]. For instance, Li et al. reported that when the Cr content was 2 at.%, the film exhibited the best microstructure density and mechanical properties, while the electrical conductivity remained at a relatively high level. Furthermore, doping with low Cr content is also more relevant in industrial applications. Because in highly conductive copper alloys, excessive Cr can lead to a significant increase in resistivity, which is not conducive to the usage requirements of electrical contact materials [8]. Therefore, this study focuses on the range of 1–3 at.% to systematically evaluate the effects of different low Cr contents on the microstructure, mechanical properties, and corrosion resistance of Cu films.

At present, studies on the preparation of high-performance Cu-Cr alloy films on BaTiO_3_ ceramic substrates using HiPIMS technology have not been reported. This study aims to utilize the high ionization characteristics of HiPIMS to prepare copper films and copper–chromium alloy films with Cr contents of 1 at.%, 2 at.%, and 3 at.% on the BaTiO_3_ substrate at room temperature, and explore the influence of different Cr contents on the microstructure and properties of Cu films. It aims to prepare Cu-Cr films with high electrical conductivity, strong adhesion and excellent corrosion resistance.

## 2. Experimental Details

### 2.1. Sample Preparation

Cu-Cr alloyed films with different Cr volume concentrations (0, 1, 2, 3 at.%) were fabricated on 10 mm × 10 mm × 0.5 mm (100) Si provided by Quanzhou Qijin New Material Technology Co., Ltd. (Quanzhou, China) and 10 mm × 10 mm × 0.5 mm BaTiO_3_ ceramic substrates provided by Beijing CAS Yannuo New Material Technology Co., Ltd. (Beijing, China) by high-power pulsed magnetron sputtering technology. Alloyed films with a thickness of approximately 1.5 μm were fabricated using Cu (99.999% purity, 50.3 mm diameter, 3 mm thickness), Cu-1 at.% Cr, Cu-2 at.% Cr, and Cu-3 at.% Cr targets provided by Deyang ONA new materials Co., Ltd. (Deyang, China). Prior to vacuum chamber placement, the sample was cleaned using both an alcohol bath and an ultrasonic method. All depositions were conducted using a magnetron sputtering system (Model DTS-200) featuring a rectangular vacuum chamber with three magnetron cathodes. One cathode was equipped with a circular target measuring 50.3 mm in diameter and 3 mm in thickness, with a purity of 99.99%. Prior to deposition, the chamber was evacuated in two stages: first to a rough vacuum below 10 Pa using a mechanical pump, followed by a high vacuum of 3 × 10^−3^ Pa achieved with a turbomolecular pump. An unbalanced magnetic field was generated by the magnetron above the copper target to enhance electron confinement. The target was then energized by a high-power pulse power supply, which operated in a constant voltage mode and delivered a pulsed negative bias of −500 V. In situ Ar ion cleaning was performed at a substrate bias of −500 V for 5 min immediately before deposition to remove any residual surface layers. For this research topic, the pulse length process parameters of HiPIMS technology were 100 µs, the constant frequency was 300 Hz, the corresponding duty cycle was 3%, the average power was 180 W and the peak current was 14 A. Throughout the deposition process, the chamber pressure was stabilized at 0.6 Pa. High-purity argon gas (Ar, 99.999%) was introduced at a constant flow rate of 100 sccm, regulated by a mass flow controller. A summary of the detailed deposition parameters is provided in Table 1.

### 2.2. Characterization

The crystal structure was analyzed by an X-ray diffractometer provided by Rigaku SmartLab (Tokyo, Japan) (9 KW) in grazing incidence mode (GI-XRD). X-ray diffractograms of copper thin films deposited on silicon wafers were acquired using a Cu Kα source (λ = 1.5406 Å) at an angle of 40° to 100° with the following measurement parameters: ω = 0.5°, step = 0.02°, speed = 4°/min, incident slit = 0.5 mm. The surface morphology and cross-sectional thickness of the films deposited on silicon wafers were characterized using a field-emission scanning electron microscope, model Gemini SEM 560, provided by Carl Zeiss AG (Oberkochen, Cermany). Five cross-sections of the samples were selected by SEM, and the cross-sections of each sample were measured five times. The arithmetic mean was taken to obtain the thickness of the copper film. The surface elements and content distribution were tested by scanning electron microscope (Gemini SEM560) combined with energy dispersive spectrometer (EDS). Elemental composition of the Cu and Cu-Cr alloy thin films was characterized by EDS using both point analysis and area mapping (scan areas 10–100 µm^2^) with an accelerating voltage of 10 kV and a working distance of 8.5 mm. Five points were taken from each sample for testing and the average value was calculated. The three-dimensional microscopic morphology and roughness of the copper film deposited on the silicon wafer surface were characterized with the probe tapping mode using atomic force microscopy provided by Bruker Nano Inc. in the Billerica, MA, USA, where the roughness values were obtained by taking multiple 1 µm × 1 µm regions within a range of 5 µm × 5 µm, and then the arithmetic mean of multiple sets of roughness values was taken. The square resistance of the copper film deposited on BaTiO_3_ was evaluated using the four-probe tester (RTS-9), and the film’s resistivity was calculated in combination with the measured thickness. Five points were taken along the diagonal of the thin film sample for measurement, and then the arithmetic mean was taken to obtain the square resistance value. Film–substrate adhesion strength of thin film deposited on BaTiO_3_ was evaluated by scratch testing, employing a diamond indenter (conical/hemispherical tip) drawn across the surface at constant velocity while applying a linearly increasing normal load. The critical load for film delamination determined adhesion strength. All scratches were performed under maximum load 50 N, loading rate 25 N/min, and scratch length 5 mm.

Prior to electrochemical measurements, the samples were secured to copper wire leads with insulating tape and positioned at the bottom of the test tube, leaving 1 cm^2^ of their surface in contact with the solution. The Gamry Interface 1010E electrochemical workstation, provided by Gamry Instruments (Warminster, PA, USA), was employed for electrochemical tests, using a standard three-electrode system: the sample as the working electrode, a stone grinding rod as the counter electrode, and a saturated Ag/AgCl electrode as the reference. Electrochemical experiments were carried out under open-circuit potential stability. Potentiodynamic polarization was measured from −500 mV to 0 mV (SCE) with a scan rate of 1 mV/s. The polarization curve was analyzed by the Tafel extrapolation method. In the frequency range of 100 kHz to 0.01 Hz, with an alternating voltage amplitude of 5 mV, electrochemical impedance spectra were tested, and 10 data points could be obtained for each order of magnitude. The electrochemical impedance spectra were analyzed using ZView software (version 2.7).

## 3. Results and Discussion

### 3.1. Microstructure and Morphology of Cu and Cu-Cr Alloyed Thin Films

**Figure 1:**
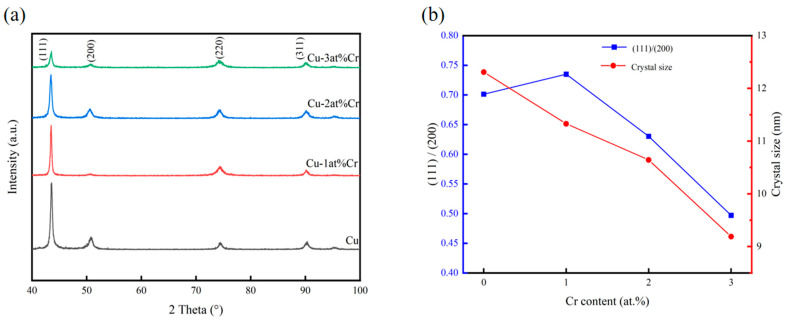
Effect of Cr content on the structure of Cu-Cr films: (**a**) XRD patterns; (**b**) evolution of grain size and the (111)/(200) peak intensity ratio.

Figure 1 shows the XRD diffraction patterns, grain sizes, and peak intensity ratios of (111)/(200) of all samples within the range of 40–100°. It can be seen that the films were all polycrystalline structures with sharp diffraction peak shapes, and the films had good crystallinity. The preferred orientations of copper films are generally (111), (200), and (220), among which the surface energy of the (111) crystal plane is the lowest. The free energy of film growth is composed of strain energy and surface energy. During the film deposition process, it grew in the direction of reducing its own free energy. When the growth of a thin film is not affected by stress, it generally grows along the (111) crystal plane. When subjected to stress, the strain energy and surface energy jointly control the crystal plane orientation of the copper film. When the strain energy is relatively high, in order to reduce the growth of free energy of the system, the copper film will grow towards the (200) crystal plane with the lowest strain energy [30,31,32]. It can be seen from the figure that after adding Cr, the structure of the copper–chromium alloy film had not changed, and its diffraction peak position was consistent with that of the pure metal copper film. The phase of the surface of the copper–chromium alloy film had not changed. The relative strength of the crystal planes and the variation in grain size of the copper-chromium alloy films (111) with different Cr contents prepared are presented in Figure 1b. The relative strength of the (111) crystal plane was calculated from (111) relative intensity = (111) intensity/(200) strength. It can be found that when the content is 1 at.% Cr, the relative strength of the (111) crystal plane increased. However, with the increase in Cr content, the relative strength of the (111) crystal plane gradually decreased, indicating that with the increase in Cr content, the orientations of other oriented crystal planes such as the (200) and (220) crystal planes increased. This was obviously the result of the interaction between surface energy and strain energy. With the increase in Cr content, the diffraction peaks did not undergo significant systematic shifts, indicating that the film had a relatively low internal stress level. It can be clearly observed from Figure 1a that as the doping amount of Cr increased, the diffraction peaks broadened significantly. According to the Scherrer formula, the microcrystalline sizes of the films prepared with different Cr contents were estimated. The Scherrer formula [33] is as follows:

(1) $$D\;=\;\frac{K\gamma }{Bcos\;\theta }$$

where *D* is the grain size, *K* is the Scherrer constant, *B* is the half-peak width or integral width of the diffraction peak of the sample being measured, *θ* is the Bragg Angle, and *γ* is the wavelength of the X-ray. It was found that with the increase in Cr content, the grains of Cu-Cr alloy films gradually decreased, indicating that the chromium element doped in the copper films played a role in refining the grains [7,29]. At room temperature, copper and chromium are almost immiscible. When chromium in the copper–chromium alloy target is sputtering down and deposited on the substrate, its lattice structure, different from that of copper, is prone to causing lattice distortion of the copper and hindering the further growth of surrounding copper grains, promoting the nucleation of grain boundaries in the film. Therefore, the grains of the copper–chromium alloy film are relatively smaller than those of the copper film. This is regarded as the result of the reduced mobility of surface and bulk atoms during the thin film deposition process and the nucleation of repetitive grains [34]. Due to the low chromium content in the copper–chromium alloy film, the presence of chromium cannot be effectively observed in the XRD diffraction spectrum. Subsequently, the existence of Cr was proved by EDS.

**Figure 2:**
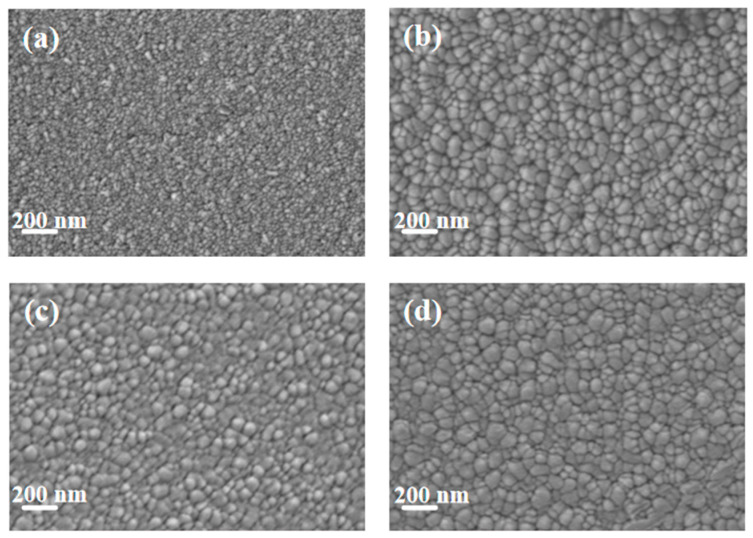
SEM Surface morphology of thin films under different Cr content conditions: (**a**) Cu; (**b**) Cu-1 at.% Cr; (**c**) Cu-2 at.% Cr; (**d**) Cu-3 at.% Cr.

Figure 2 shows the SEM images of pure Cu films and copper–chromium alloy films with varying Cr content doping conditions. It can be found that the surface of the pure Cu film without Cr doping presented a uniform granular structure with small particle size and dense distribution. After Cr element doping, compared with the pure Cu film, the particles on the film surface showed a coarsening phenomenon. This can be explained by the fact that under the bombardment of high-energy particles, Cr may preferentially segregate towards the surface, reducing the surface energy of Cu [7]. Atoms diffuse through the surface and transfer from small particles with high surface energy to large particles with low surface energy, resulting in the dissolution of small particles and the growth of large particles [35]. It is worth noting that the SEM here displays the geometric dimensions of “surface particles”, which were significantly influenced by surface diffusion and thermodynamic processes. The “bulk crystal domain size” obtained through XRD peak broadening analysis (Figure 1) reveals the integrity within the crystal. There were no obvious holes on the surfaces of any of the samples, and the surfaces all had good density. It can be observed from the figure that compared with other Cr content films, when the Cr content was 2 at.%, the surface particles of the film were relatively smaller and the film was denser.

**Figure 3:**
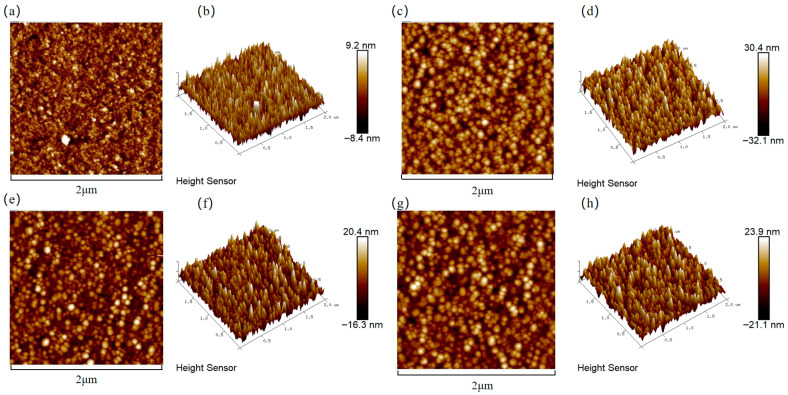
Cr content-dependent surface topography of Cu films analyzed by AFM: (**a**,**b**) Cu; (**c**,**d**) Cu-1 at.% Cr; (**e**,**f**) Cu-2 at.% Cr; (**g**,**h**) Cu-3 at.% Cr.

**Figure 4:**
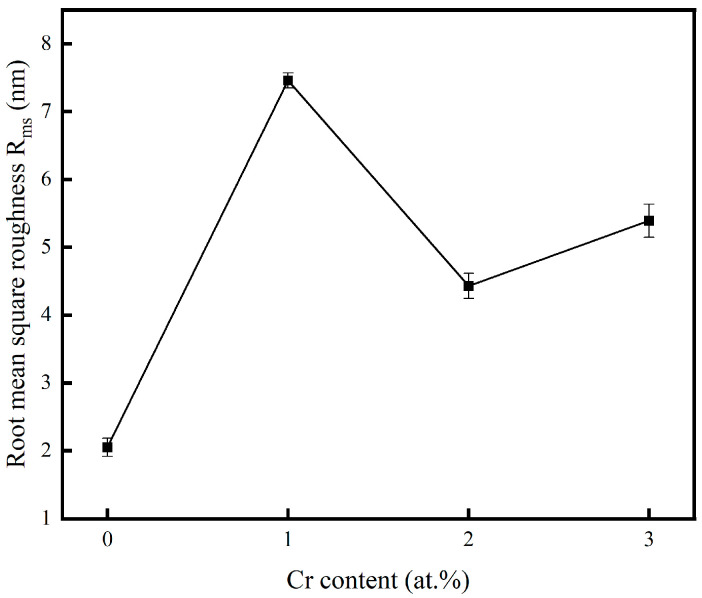
Variation in root mean square roughness.

Figure 3 presents the surface roughness of the copper films, measured by AFM, across varying Cr contents. The incorporation of chromium into the copper matrix resulted in a significant increase in surface roughness compared to pure Cu films. The increase in surface roughness was attributed to the coarsening of surface particles, as clearly evidenced by the SEM micrographs. To visualize the relationship between the surface roughness of the copper film and the Cr content, Figure 4 shows the trend graphs of the root mean square roughness of the sample surface under different Cr contents. The observed rise in surface roughness with higher Cr content can be fundamentally explained by Ostwald ripening, a thermodynamically driven process in which larger surface features grow at the expense of smaller, less stable ones, leading to overall particle coarsening [35]. The surface roughness of the film is presented in Table 2. When the doping amount was 2 at.%, compared with the pure Cu film, Ra = 2.05 nm increased to Ra = 4.43 nm. At this point, the doped film still maintained a relatively low surface roughness.

**Figure 5:**
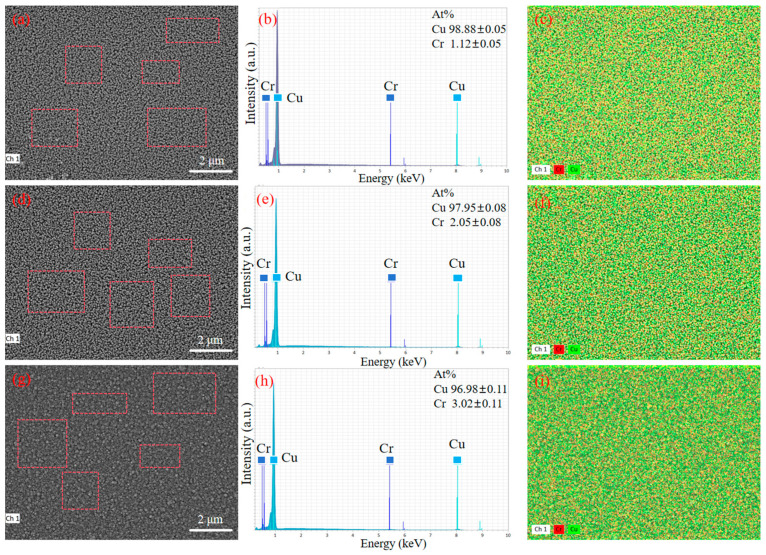
SEM images and corresponding EDS pattern of the copper–chromium alloy film: (**a**–**c**) Cu-1 at.% Cr; (**d**–**f**) Cu-2 at.% Cr; (**g**–**i**) Cu-3 at.% Cr.

Figure 5 shows the EDS scanning images of the surface of copper–chromium alloy films with different Cr contents. From the figure, the thin film of the average content of Cr is (1.12 + 0.05) at. %, (2.05 − 0.08) at. %, and (3.02 + 0.11) at.%, slightly higher than the design target of 1 at.%, 2 at.% and 3 at.%. The lower standard deviation and the uniform surface distribution of Cr elements shown in Figure 5b,c,e,f,h,i jointly prove that the distribution of Cr elements is uniform at the micrometer scale, and no macroscopic segregation is observed. The spatial resolution of EDS is limited (usually at the micrometer level), and it may not be able to detect grain boundary segregation or tiny precipitated phases at the nanoscale [36]. As can be seen from Figure 1, no diffraction peaks belonging to the second phase such as pure Cr or Cu-Cr compounds were observed in the XRD pattern. This indicates that even if there is nano-scale Cr segregation, its size and volume fraction are still below the detection limit of XRD [36], which indirectly supports the view that most Cr atoms may exist in the form of solid solutions.

### 3.2. Performance

The thickness, sheet resistance, and resistivity of the thin films are shown in Table 3.

**Figure 6:**
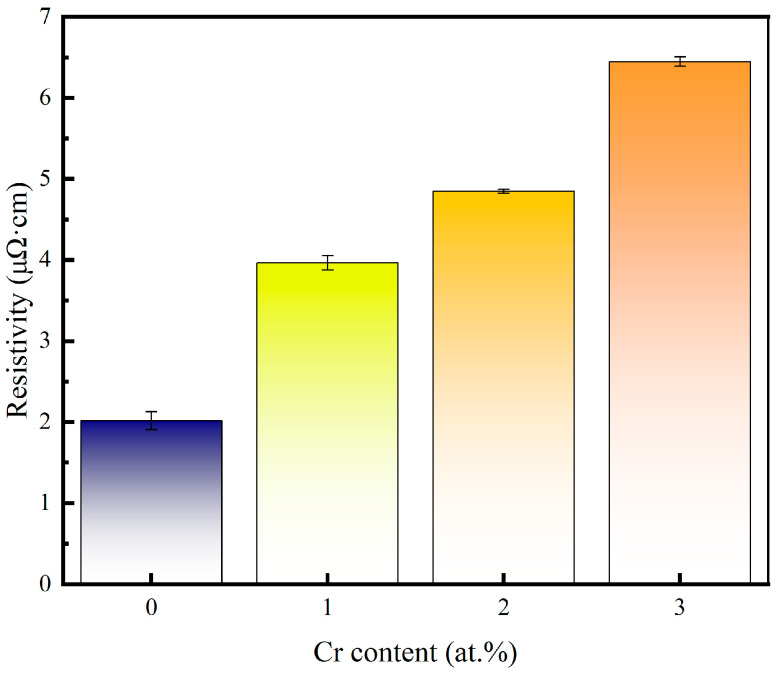
Variation in copper film and copper–chromium alloy film resistivity versus Cr content.

The resistivity of the pure copper film and the Cu-Cr alloy film doped with Cr is shown in Figure 6. Compared with pure copper films, the resistivity increases with the increase in Cr content after Cr doping. Grain refinement and the doping of alloying elements in the grains are the main reasons for the increase in the resistivity of alloy films. According to Matthiessen’s Rule, the resistance of a metal is composed of its fundamental resistance and additional resistance caused by solute atoms, lattice defects, etc. Therefore, under the same preparation process as copper films, the increase in the resistivity of copper–chromium alloy films is mainly caused by the lattice defects of the films resulting from the solute atom chromium and chromium doping [37]. For copper–chromium alloy films, chromium, as a solute atom during the sputtering process, causes lattice distortion of copper, resulting in a large number of defects inside the film and, thus, causing the copper–chromium alloy film to have a relatively high resistivity. Meanwhile, after doping with Cr, the grain size decreases and the grain boundary area in the copper–chromium alloy film increases, which also has a strong scattering effect on the movement of electrons [7]. Moreover, a higher surface roughness contributes more to the interfacial/surface scattering of electrons [38]. Therefore, the high Ra of Cu-1 at.% Cr thin films can also explain their higher resistance, while Cu-2 at.% Cr film achieves grain refinement and density improvement while maintaining a relatively low Ra, which corresponds to the previous AFM results.

In the Ni/Cr co-doping study (taking annealing at 500 °C as an example), the resistivity of the film with a Cr content of 1 at.% was approximately 4.5 μΩ·cm [39], which was comparable to 3.97 μΩ·cm under the conditions of room temperature deposition without annealing. This indicates that without high-temperature densification/grain growth, low Cr content will bring about an increase in resistivity of the same magnitude as that in the literature. Higher doping (≈3 at.% Cr) usually leads to a more significant increase in resistivity: our sample was 6.45 μΩ·cm at 3 at.% Cr, which is consistent with the reported trend of a significant increase in film resistivity caused by Cr doping. Furthermore, the Cu-0.3% Cr-0.2% Zr film containing extremely small amounts of Cr and Zr can be reduced from 4.80 to 2.96 μΩ·cm after annealing at 300 °C/1 h, which also indicates that heat treatment and defect control can improve electrical conductivity [40]. This quantitative comparison indicates that the variation in our resistivity mainly results from the combined effect of the increase in doping content and the defects in the thin-film state, rather than an accidental outcome caused by individual experimental conditions.

**Figure 7:**
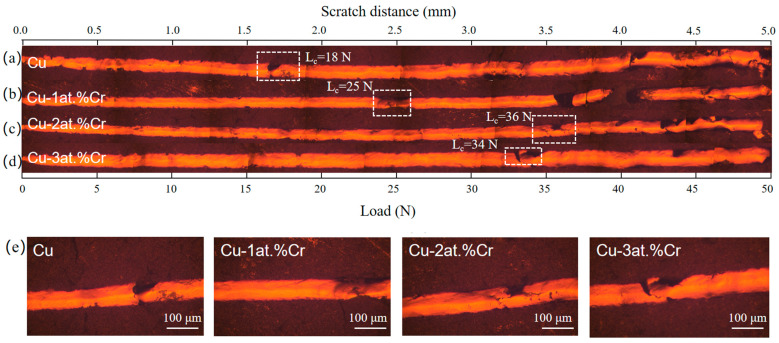
Scratch morphologies of Cu films with different Cr contents: (**a**) Cu; (**b**) Cu-1 at.% Cr; (**c**) Cu-2 at.% Cr; (**d**) Cu-3 at.% Cr; (**e**) Scratch morphology at the critical load of the film.

Figure 7 shows the scratch morphology of Cu films with different Cr contents on BaTiO_3_ ceramic substrates, and the critical load L_c_ obtained from the scratch morphology. All scratch tests were conducted using linearly increasing loads, with a maximum of 50 N at a scratch length of 5 mm. The critical load (L_c_) is determined by correlating the scratch morphology characteristics with the load force curve. It can be observed that the critical load of the pure Cu film is the lowest, with L_c_ = 18 N. The critical loads of Cu-Cr films with Cr contents of 1 at.%, 2 at.%, and 3 at.% are 25 N, 36 N, and 34 N, respectively. The specific changing trends are shown in the figure. It indicates that the addition of Cr content enhances the film–base adhesion between the Cu film and the ceramic substrate. This is mainly due to the addition of trace amounts of Cr, which forms local Cr-O bonds at the interface, increasing the chemical bond between the Cu film and the ceramic substrate. Moreover, it can be observed from the XRD analysis results that the addition of Cr refines the Cu grains, making the film denser, reducing defects, and thereby enhancing the bonding strength. The peak value is reached when the Cr content is 2 at.%. Further increasing the Cr content may lead to a decrease in binding force due to Cr segregation at the grain boundaries and interfacial brittleness [7,29].

### 3.3. Corrosion Resistance of Cu-Cr Alloy Films

#### 3.3.1. Static Corrosion of Thin Films

**Figure 8:**
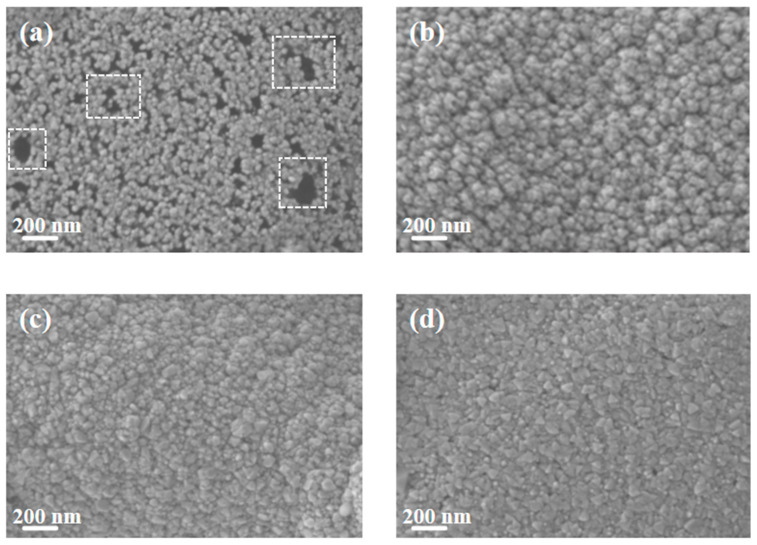
SEM morphology of the film surface after soaking in 3.5 wt% NaCl solution for 2 h: (**a**) Cu; (**b**) Cu-1 at.% Cr; (**c**) Cu-2 at.% Cr; (**d**) Cu-3 at.% Cr.

The Cu film and Cu-Cr alloy films with different Cr contents were immersed in 3.5 wt% NaCl solution for 2 h, and their surface morphologies were observed. The static effect of NaCl solution on them was studied. The surface morphologies are shown in Figure 8. It can be seen from Figure 8a that after the copper film was corroded in the NaCl solution, black granular substances and a small number of corrosion holes appeared on its surface, which are insoluble corrosion products produced after the copper was corroded in the NaCl solution. Compared with the pure copper film, it can be seen from Figure 8b–d that there are a small number of dark-colored corrosion areas on the surface of the Cu-Cr alloy film, but there is no obvious adhesion of corrosive substances or the formation of corrosion holes, indicating that it has better anti-corrosion performance in static corrosion. Compared with Figure 2b–d, after corrosion, it can be observed from Figure 8b–d that the surface particles are smaller and the edges are sharper. This is mainly because Cr atoms may have segregation at the grain boundaries, and the atoms in these regions are preferentially corroded and dissolved due to their high energy and high activity [41]. Preferred corrosion at grain boundaries disrupts the connections between surface grains, causing larger aggregates composed of multiple crystal domains to separate into smaller, independent particles after corrosion. This is manifested macroscopically as “particle reduction” [42]. Meanwhile, the corrosion process exhibits anisotropy. In regions with smaller curvature radii such as the edges and corners of particles, the mass transfer rates of reactants and products are faster, resulting in corrosion rates at these locations being much higher than those on flat crystal planes. This preferential etching effect effectively sharpened the edges of the particles [43]. However, one limitation of this study lies in the fact that the chemical composition of the corrosion products on the surface of the copper/copper–chromium alloy film after corrosion was not identified.

#### 3.3.2. Electrochemical Corrosion of Thin Films

**Figure 9:**
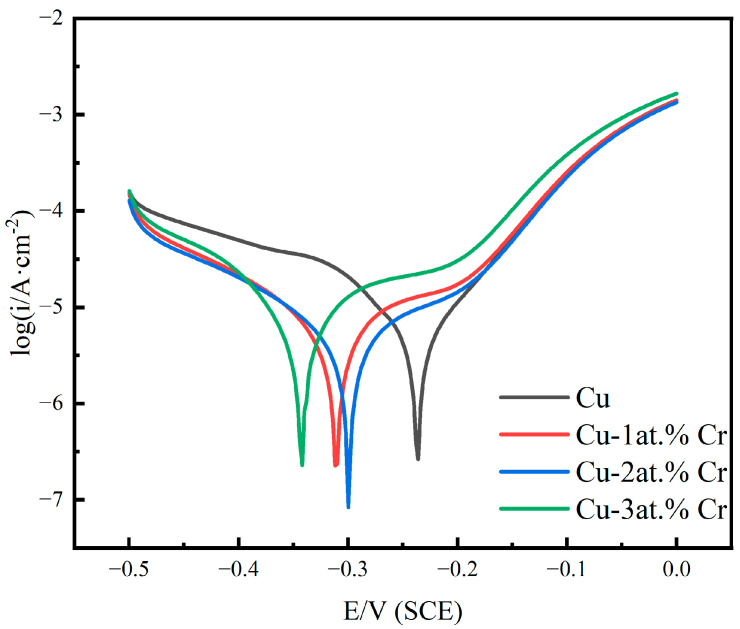
Polarization curves of copper films with different Cr contents in 3.5 wt% NaCl solution.

Figure 9 shows the polarization curves of pure Cu films and Cu-Cr alloy films with different Cr contents in 3.5 wt% NaCl solution. It can be seen that with the increase in Cr content, passivation behavior gradually occurs [44]. With the increase in Cr content, the corrosion sites move towards negative values. The results show that the Cu-Cr alloy film has a higher corrosion tendency under Cl^−^ corrosion, which may be related to the increase in the surface roughness of the alloy and the increase in the adsorption active sites of oxygen or chlorine [38,45]. The alloy exhibited a gradual reduction in corrosion current density with increasing Cr content, an effect primarily attributed to the development of a protective passive layer [45]. After doping with Cr, the corrosion current density was lower than that of the pure copper film, indicating that the corrosion resistance of the film was improved after doping with Cr. The passivation zone of copper–chromium alloy films is relatively short, and the passivation phenomenon is not obvious in a NaCl solution. At the same electrode potential, the anodic polarization current density of copper–chromium alloy films is higher than that of copper films, but its cathodic polarization current density is lower than that of copper films. In the polarization curve, the cathodic reaction of copper and copper–chromium alloy films is an oxygen diffusion-controlled reduction reaction [46]. The lower cathodic polarization current density of copper–chromium alloy films indicates that the electron loss reaction of oxygen is suppressed [47]. For copper–chromium alloy films, the addition of chromium elements refines the film grains and increases the grain boundary area, providing a large number of diffusion channels for Cl^−^ and accelerating the transport rate of Cl^−^ [48], enabling Cl^−^ to diffuse rapidly at a relatively low overpotential. Secondly, due to the relatively low cathodic reaction rate of the copper–chromium alloy film, the concentration of OH^−^ in the solution is relatively low. Therefore, the copper–chromium alloy film is damaged by Cl^−^ before it can be effectively passivated by OH^−^ in the solution [49]. Consequently, the passivation behavior is less pronounced, exhibiting a relatively high passivation current density.

Table 4 summarizes the electrochemical characteristic parameters, including the anode Tafel slope b_a_, the cathode Tafe slope b_c_, the corrosion potential E_corr_, the corrosion current density I_corr_, and the polarization resistance R_p_. As evidenced by the data in the table, the copper–chromium alloy film exhibits an increased cathodic Tafel slope relative to the pure copper film. Furthermore, the polarization resistance (R_p_) demonstrates a positive correlation with chromium content. This indicates that the addition of Cr affects the cathode reaction of the copper–chromium alloy film in NaCl solution, reduces the dissolution rate of the film, and improves the corrosion resistance [50,51].

#### 3.3.3. AC Impedance Test of Thin Films

**Figure 10:**
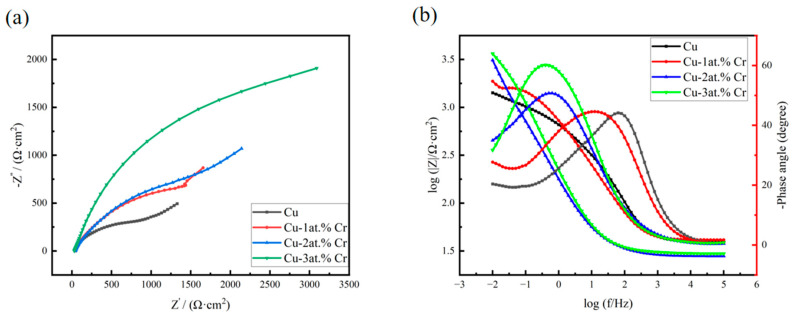
Impedance spectra of copper films with different Cr contents after being exposed in 3.5 wt% NaCl solution: (**a**) Nyquist spectra; (**b**) Bode spectra.

Figure 10 shows the AC impedance spectra of copper films with different Cr contents after exposure in 3.5 wt% NaCl solution. Figure 10a shows the Nyquist plots of copper and copper–chromium alloy films. It can be seen from the figure that the shapes of the Nyquist plots of copper and copper–chromium alloy films are the same, indicating that the corrosion mechanism of the films in 3.5 wt% NaCl has not changed [52]. It can be seen from the figure that the Nyquist curve in the high-frequency region presents as a capacitive reactance arc, which is caused by the “diffusion effect” resulting from the roughness and unevenness of the thin-film electrode surface. In the EIS test under the same conditions, the larger the capacitive reactance arc radius of the Nyquist curve is, the smaller the W diffusion region is, and the stronger the corrosion resistance of the electrode material is [53]. The diameter of the high-frequency semicircle increases with higher Cr content, indicating a corresponding rise in both the charge transfer resistance and the oxide film resistance of the thin film. Meanwhile, the Warburg impedance appears in the low-frequency region, which indicates that the corrosion mechanism is controlled not only by the charge transfer step but also by the diffusion process [54]. Figure 10b shows the Bode plot of the thin film. From the variation in the Bode plot within the frequency range, it can be seen that there are two time constants for the thin film electrode, indicating that there are two reaction mechanisms for the corrosion reaction of the thin film in NaCl solution [55]. Among them, the time constant in the high-frequency region of the Bode diagram corresponds to the reaction of the oxide on the film surface at the metal/oxide interface to form Cu^+^, and under the action of the electric field, Cu^+^ is oxidized to Cu^2+^ and migrates from the metal/oxide interface to the oxide/solution interface. The time constant in the low-frequency region of the Bode diagram corresponds to the dissolution of the metal electrode. The Cu_2_O and CuO films formed by the oxidation of the copper surface can resist the erosion of the NaCl solution to a certain extent [56], but their corrosion resistance is poor. The process of substance diffusion into the solution when the oxide film on the electrode surface corresponding to the Weber diffusion W is destroyed at this time, in addition to the charge transfer rate affecting the reaction process of the thin-film electrode in the NaCl solution, the diffusion rates of dissolved oxygen and Cl^−^ in the solution, and substances such as OH^−^, Cu^+^, and CuCl^2−^ generated by the reaction, also simultaneously affect the corrosion rate of the film [57]. In the Bode diagram, when the impedance value |Z| suddenly decreases with frequency variation, it indicates that the oxide film on the surface of the thin film electrode is damaged, and at this time, the NaCl solution destroys the interior of the film [58]. It can be seen from the Bode diagram that the copper–chromium alloy film has a relatively large impedance value [59]. According to the corresponding Bode phase angle plot, the maximum phase angle peak shifts toward lower frequencies as the Cr content increases. The increase in the maximum phase angle suggests a reduction in the corrosion rate of the alloy film with higher Cr content [60].

**Figure 11:**
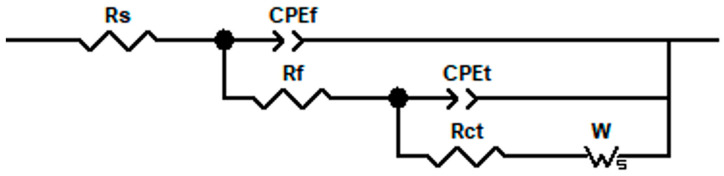
Physical models and corresponding equivalent circuits used for fitting the impedance data of copper films with different Cr contents in 3.5 wt% NaCl.

The AC impedance data of copper and copper–chromium alloy films were fitted and analyzed. The equivalent circuit is shown in Figure 11, and the EIS data fitting is basically consistent. Due to the diffusion effect in the high-frequency region of the Nyquist diagram, the offset between the double-layer capacitance on the electrode surface and the ideal pure capacitance is reflected by a CPE element in the equivalent circuit [61]. The impedance magnitude of CPE is Z_CPE_ = Q^−1^(jω)^−n^, where Q and n are constants (0 < n < 1) [62]. n is usually used to describe the non-ideal capacitive behavior on the electrode surface. Q_f_ is the pseudo capacitance of the oxide film on the surface of copper and copper–chromium alloy thin films, and R_f_ is its resistance. Q_t_ is the pseudo capacitance of the electrode surface, and W is the Weber impedance related to the diffusion of the substance, with a magnitude of Z_W_ = W_R_tanh(iTω)^n^/(iTω)^n^,0 < n < 1. In the formula, T is related to the diffusion characteristics of the electrode. Rs represents the resistance of the test solution, and R_ct_ is the charge transfer resistance of the electrode reaction, whose magnitude reflects the ease or difficulty of the reaction of the test electrode in the solution. In the same AC impedance test system, the larger the R_ct_, the more difficult the electrode reaction in the solution and the better the corrosion resistance of the film [55,63].

Table 5 shows the fitting characteristic parameters of the impedance spectrum. The data presented in the table reveal that R_ct_ exceeds R_f_, suggesting that the electrochemical reaction is predominantly governed by charge transfer processes. Furthermore, compared with the pure copper film, after Cr doping, the R_ct_ of the Cu-Cr alloy film is greater than that of the Cu film. In the same AC impedance test system, the larger the R_ct_, the more difficult the electrode reaction in the solution and the better the corrosion resistance of the film, indicating that the addition of Cr can improve the corrosion resistance of the film. Moreover, with the increase in Cr content, R_f_ also increases, indicating that as the Cr content increases, the oxide film on the outer layer of the film becomes dense, effectively blocking the diffusion of substances and improving the structure of the corrosion product film [62]. Therefore, the incorporation of Cr enhances the density of the oxide layer, leading to a decreased corrosion rate and improved corrosion resistance of the alloy film in NaCl solution. And it can be seen from W_R_ that the copper–chromium alloy film has a higher diffusion impedance than the copper film. Therefore, doping chromium in copper affects the transport of substances related to electrode reactions in the solution [64], which is consistent with the fact that the copper–chromium alloy film has a higher cathode Tafel slope than the copper film in polarization tests. Chromium reduces the cathode reaction rate of the copper film in NaCl solution. CPE_f_ represents the double-layer characteristics of the oxide layer on the surface of the electrode film in the solution [65], and Q_f_ and n_f_ are parameter indicators indicating its impedance magnitude. It can be seen from Table 5 that there are significant differences in Q_f_ and n_f_ between copper and copper–chromium alloy films, indicating that the surface oxide layers of copper–chromium alloy films and copper films are different, and the surface of the films changes after chromium blending. Compared with pure Cu films, the n_f_ of the film show a downward trend and the Q_f_ increases after adding a trace amount of Cr, indicating that the non-ideality of the film is enhanced, the surface roughness is improved, the uniformity of the film decreases, and the density of the film decreases [62]. Additionally, the n_f_ of the Cu-Cr film doped with 2 at.% Cr is relatively large, and the Q_f_ is relatively small, indicating that the surface of the film at this time is still relatively dense and uniform compared with other doping amounts. This is consistent with the SEM and AFM analysis results mentioned earlier.

With the addition of Cr element, n_t_ shows an upward trend, indicating that Cr makes the interface between the electrode and the solution more uniform. Among them, the Cu-Cr film doped with 2 at.% Cr has the largest n_t_. Therefore, when the addition amount is 2 at.% Cr, it can effectively enhance the corrosion resistance of the film and ensure its density.

## 4. Conclusions

In this study, the influence of different Cr doping amounts on the structure and properties of Cu thin films was investigated.

(1) Compared with pure Cu films, after Cr doping, as the Cr content increases, the lattice structure different from that of copper is prone to cause lattice distortion of the copper, hindering the further growth of surrounding copper grains, resulting in the gradual reduction in film grains. The surface particles show a coarsening phenomenon due to the influence of surface energy, but they are still relatively dense.
(2) Compared with pure Cu films, after doping with Cr, the resistivity increases with the increase in Cr content. Grain refinement and doping of alloying elements in the grains are the main reasons for the increase in the resistivity of alloy films. In addition, an appropriate amount of Cr doping helps to enhance the film–base binding force between the Cu film and BaTiO_3_.
(3) Compared with pure Cu films, the films doped with Cr have better corrosion resistance in static corrosion. The addition of Cr affected the cathodic reaction of the copper–chromium alloy film in NaCl solution, reduced the dissolution rate of the film, and improved the corrosion resistance. Compared with the pure copper film, after the addition of Cr, the oxide film resistance and the charge transfer resistance of the electrode reaction of the Cu-Cr alloy film were greater than those of the Cu film, indicating that the addition of Cr reduced the corrosion rate of the alloy film and improved the corrosion resistance of the film in NaCl solution.

For metallized dielectric filters, it is generally required that the resistivity of the metal layer be lower than 5 μΩ·cm to minimize insertion losses [5], while the adhesion strength to the ceramic substrate should exceed 30 N to ensure long-term reliability in damp or saline environments [3,23]. Comprehensively consider the adhesion between the film and the substrate, the conductivity, and the results of electrochemical tests. In our research, the resistivity of the 2 at.% Cr film was 4.85 μΩ·cm, which was completely within the acceptable range. The adhesion strength was 36 N, exceeding the application threshold. Moreover, the electrochemical indicators were good, and the charge transfer resistance was nearly twice that of the pure Cu film. In contrast, although the pure Cu film has a relatively low resistivity (2.01 μΩ·cm), its adhesion is insufficient (18 N), and although the 3 at.% Cr film has better corrosion resistance, its resistivity is 6.45 μΩ·cm, which is higher than the typical application limit. Therefore, when the Cr addition amount is 2 at. %, it balances the film–base bonding force, electrical conductivity, and required corrosion resistance. The combination of adhesion, resistivity, and corrosion performance observed here is promising with respect to dielectric filter applications, but further long-term reliability and system-level integration studies are required before industrial requirements can be claimed.

## Figures and Tables

**Table 1:** Details and parameters of sputtering.

Parameters	HiPIMS (Sample 1/2/3/4)
Target to substrate distance (mm)	75
Substrate temperature (°C)	36
Working pressure (Pa)	0.6
Substrate rotation speed (r/min)	8
Total deposition time (min)	45
Pulse frequency (Hz)	300
Pulse width (μs)	100
Cr content (at.%)	0/1/2/3
Pulse-negative voltage (V)	500
Peak discharge current (A)	14
Average power (W)	180
Bias voltage (V)	100

**Table 2:** The surface roughness of the film was measured by AFM (mean ± SD, *n* ≥ 5).

Parameters	Cu	Cu-1 at.% Cr	Cu-2 at.% Cr	Cu-3 at.% Cr
Rq (nm)	2.31 ± 0.21	9.32 ± 0.36	5.28 ± 0.22	6.12 ± 0.13
Ra (nm)	2.05 ± 0.13	7.46 ± 0.11	4.43 ± 0.18	5.39 ± 0.25

**Table 3:** The thickness, sheet resistance, and resistivity of the thin films (mean ± SD, *n* ≥ 5).

Parameters	Cu	Cu-1 at.% Cr	Cu-2 at.% Cr	Cu-3 at.% Cr
Thickness (nm)	1468 ± 32	1513 ± 28	1489 ± 54	1544 ± 25
Sheet Resistance (Ω/□)	13.7 ± 1.1	26.25 ± 1.2	32.6 ± 1.3	41.8 ± 1.1
Resistivity (μΩ·cm)	2.01 ± 0.11	3.97 ± 0.12	4.85 ± 0.02	6.45 ± 0.06

**Table 4:** The characteristic parameters obtained from the polarization curves of copper films with different Cr contents in 3.5 wt% NaCl solution.

Parameters	Cu	Cu-1 at.% Cr	Cu-2 at.% Cr	Cu-3 at.% Cr
E_corr_ (V)	−0.238	−0.302	−0.324	−0.346
I_corr_ (A·cm^−2^)	8.12 × 10^−6^	6.59 × 10^−6^	4.46 × 10^−6^	1.02 × 10^−6^
b_a_ (V·dec^−1^)	14.11	3.55	5.71	2.48
b_c_ (V·dec^−1^)	4.68	6.21	6.15	6.11
R_p_ (Ω·cm^2^)	1852.6	4592.3	5823.3	7678.1

**Table 5:** Elements of the equivalent circuit for copper films with different Cr contents after being exposed in 3.5 wt% NaCl solution.

Parameters	Cu	Cu-1 at.% Cr	Cu-2 at.% Cr	Cu-3 at.% Cr
R_s_ (Ω·cm^2^)	39.86	40.76	31.57	29.84
Q_f_ (Ω^−1^·cm^−2^·S^n^)	4.44 × 10^−5^	1.32 × 10^−4^	1.18 × 10^−4^	9.87 × 10^−4^
n_f_	0.86	0.77	0.77	0.76
R_f_ (Ω·cm^2^)	252.1	430.2	471.6	480.7
Q_t_ (Ω^−1^·cm^−2^·S^n^)	4.63 × 10^−4^	2.16 × 10^−4^	2.97 × 10^−4^	9.79 × 10^−5^
n_t_	0.57	0.81	0.87	0.76
R_ct_ (Ω·cm^2^)	778.2	804.5	1352	4641
W_R_ (Ω·cm^2^)	1911	2947	3976	6321

## Data Availability

The original contributions presented in this study are included in the article. Further inquiries can be directed to the corresponding author.
